# Safe and Rapid Cure for Aneurism

**Published:** 1877-07

**Authors:** 


SAFE AND RAPID CURE FOR ANEURISM.—(*Brit. Med. Journal*, Feb. 3, 1877).—Dr. Horace Dobell, submits to surgeons a simple suggestion for the cure of aneurism. It is to stop the circulation above and below the tumor, remove the fluid contents of the latter by aspiration, and replace them by an injection of spermaceti or stearin. The latter are insoluble in blood, but solid at its temperature; fluid at a temperature low enough to allow of their being safely brought into contact with living tissues, and changes from liquid to solid with great rapidity; and are at the same time light, innocuous and unirritating. Their rapid solidification removes any danger of active or passive clots being washed away when the blood is allowed again to flow, while the time for the operation would be so short, that no harm would result to the other tissues on account of the arrested circulation.—*Detroit Med. Journal.*

